# PRO-DEMET Randomized Controlled Trial on Probiotics in Depression—Pilot Study Results

**DOI:** 10.3390/nu15061400

**Published:** 2023-03-14

**Authors:** Oliwia Gawlik-Kotelnicka, Aleksandra Margulska, Anna Skowrońska, Dominik Strzelecki

**Affiliations:** 1 Department of Affective and Psychotic Disorders, Medical University of Lodz, 92-216 Lodz, Poland; 2 Department of Adolescent Psychiatry, Medical University of Lodz, 92-216 Lodz, Poland

**Keywords:** depression, metabolic syndrome, probiotics, feasibility, pilot study

## Abstract

There is a pressing need to identify new treatment options for depression and its comorbidities. Depression often coexists with metabolic complications, and the two may share a pathophysiological overlap, including inflammation and microbiota changes. Microbiota interventions (e.g., probiotics) may represent a safe and easy-to-use treatment option as an adjunctive therapy in patients only partially responsive to pharmacologic treatment. (1) Objective: The paper presents the results of a feasibility and pilot study. The study is an internal part of a randomized controlled trail (RCT) of the effect of probiotic supplementation on psychometric, anthropometric, metabolic, and inflammatory parameters in adult patients with depressive disorders depending on the presence of metabolic syndrome. (2) Methods: The trial has a four-arm, parallel-group, prospective, randomized, double-blind, controlled design. Sixty participants received a probiotic preparation containing *Lactobacillus helveticus* Rosell^®^-52 and *Bifidobacterium longum* Rosell^®^-175 over 60 days. The feasibility of the study design was assessed, as well as the rates of recruitment, eligibility, consent, and study completion. The following were assessed: depressive, anxiety and stress symptoms, quality of life, blood pressure, body mass index and waist circumference, complete blood count with differential, serum levels of C-reactive protein, high-density lipoprotein cholesterol, triglycerides, fasting glucose, some secondary markers of inflammation and metabolic health, as well as noninvasive biomarkers of liver fibrosis (APRI and FIB-4). (3) Results: The study was found to be generally feasible. The eligibility rate was 52% of recruited participants with 80% completing the study protocol. No differences in sociodemographic or anthropometric factors or basic laboratory findings were found between the placebo and probiotic group at the start of the intervention period. Importantly, the proportion of recruited participants fulfilling the criteria of metabolic syndrome was too low. (4) Conclusions: Whilst the whole study protocol was feasible, some different timepoint procedures require modification. The major weakness of the recruitment methods was that the percentage of metabolic arms participants was insufficient. Overall, the full RCT design on probiotics in depression with vs. without metabolic syndrome was shown to be feasible with little modification.

## 1. Introduction

The global incidence of depressive disorders and metabolic syndrome (MetS) is growing, and is responsible for significant morbidity and mortality [1]. Depression often coexists with metabolic abnormalities [2], and MetS is diagnosed in 30% of depressed subjects [3]. Importantly, both obesity and MetS have been found to be independently associated with depressive symptoms and inflammation [4]. Depressive disorders also appear to be associated with metabolic-associated fatty liver disease (MAFLD), formerly known as non-alcoholic fatty liver disease, a multisystem disease considered the hepatic manifestation of MetS [5,6]. This association has been attributed to some extent to atypical antipsychotics medication use, diet, and physical activity [7,8]. However, they cannot explain the full comorbidity rates, and a possible pathophysiological overlap is being considered, with chronic inflammation and dysbiosis being suggested as possible connecting factors [2,3,6,7].

The role of gut microbiota function in the pathophysiology of civilization diseases has recently become a source of interest [9]. The intestinal microbiota is believed to be an essential part of the gut–brain axis, with important roles being played by the autonomic and enteric nervous systems, the neuroendocrine and the immune system [10,11]. Interestingly, the bidirectional microbiota-gut-brain axis has been found to be involved in neuroinflammation and mental health disorders in both human and animal studies [12]. Interestingly, microbiota diversification has emerged to play a role in the occurrence of mood and anxiety disorders [13,14,15,16]. Furthermore, patients diagnosed with major depression showed altered levels of microbiota-derived metabolites, e.g., short-chain fatty acids [17]. Moreover, increasing evidence suggests that dysbiosis (imbalanced microbiota) may lead to systemic chronic inflammation [18], which may serve as a link between microbiota dysfunction and depression. Additionally, the gut microbiota plays a crucial role in fat storage and energy metabolism, and dysbiosis may play a role in the etiopathogenesis of metabolic health-related abnormalities, including abdominal obesity or MAFLD [19,20,21,22]. Overall, most studies indicate that overweight individuals, particularly those with metabolic complications, demonstrate less gut microbiota diversity than those of normal weight [23].

Microbiota interventions, e.g., those based on diet changes or consumption of prebiotics, may reduce the risk of depression [24], or MetS and its sequelae [25]. Moreover, using probiotics, i.e., “live microorganisms that, if consumed in adequate amounts, bring the host health benefits” [26] or synbiotics, i.e., probiotics given together with a specific prebiotic, may help alleviate depressiveness, anxiety and stress in healthy subjects and patients [27,28,29,30,31,32,33], and improve the well-being of patients [34]. However, the results from studies are conflicting. Probiotics may also help restore metabolic homeostasis, e.g., improve body mass index (BMI), lipid profile, and glucose metabolism [35,36,37,38,39,40,41]. Nevertheless, they seem to offer little benefit regarding metabolic abnormalities [36]. Despite this, probiotic supplementation may restore the imbalances in some inflammatory biomarkers, or alleviate the clinical signs of chronic inflammation [35,36,38,41,42,43].

Hence, there is a need to identify specific conditions, including features in the clinical population, that may support the curative action of probiotics. For example, there is limited but promising evidence with regard to the effectiveness of probiotics regarding the risk of depressiveness or anxiety in the perinatal period [44,45], and probiotics may have a favorable effect on overweight-induced cognitive impairment and anxiety [46]. More specifically, little is known whether probiotic mixtures have favorable effects on psychometric, metabolic and inflammation measures in a population with depression, with or without MetS as a comorbidity [47].

It is known that the effects of probiotic supplementation are strongly strain-dependent, and it is widely believed that a mixture of collaborating microbes would be more beneficial than a single strain. Therefore, a combination of *Lactobacillus* and *Bifidobacterium* strains were selected for the study. The supplementation of these probiotics has yielded promising but inconclusive outcomes in several clinical studies in healthy subjects and patients with depression [48,49,50,51]. In addition, *Lactobacillus* and *Bifidobacterium* species have specific effects on the inhibition of pro-inflammatory cytokines synthesis, which may aid in their antidepressant effect [52]. Moreover, the specific *Lactobacillus helveticus* Rosell^®^-52 and *Bifidobacterium longum* Rosell^®^-175 strains chosen for the study have been found to improve emotional behavior in animal models [53,54,55]. Furthermore, recent study results suggest that supplementation with *Bifidobacterium* species may improve both insulin resistance and obesity treatment efficacy [56], which is particularly important for our study design. The amount of supplemented probiotics was chosen based on recent randomized controlled trials (RCTs) in the field [51,57,58]. To avoid possible adverse effects from higher doses, the supplementation was provided as 3 × 10^9^ colony forming units (CFU).

Based on the above rationale, a Pro-demet randomized controlled trial protocol was constructed [59]. However, to ensure that the trial was feasible and to determine possible recruitment, eligibility and retention rates, an internal feasibility and pilot study was performed with an identical design to a Pro-demet full RCT.

The primary aim of this pilot study was to assess eligibility rates. The secondary aims were to assess the recruitment and enrolment rates, and the capacity and resources to conduct all trial processes; examine potential participant retention and adherence among the allocation groups, and data completeness. Several tertiary aims are also discussed in the manuscript.

Our hypothesis was that the recruitment rate would be around 15–20 subjects per month and that at least half of them would be eligible and would agree to enroll.

This pilot study manuscript has been planned and prepared according to a checklist for pilot studies using the CONSORT statement [60,61].

## 2. Materials and Methods

### 2.1. Design

The Pro-demet pilot trial described herein is an internal feasibility and pilot study included as part of the research design of a larger main study. It was designed as a single-center, parallel-group, prospective, randomized, double-blind, placebo-controlled pilot trial. It took place at the Medical University of Lodz (Poland).

Adult patients (≥18 years) with depressive disorders (as defined by 11th International Classification of Diseases [62]) were randomly assigned (1:1) into groups via computer-generated blocked lists stratified by the presence of MetS. Each participant received 60 days of treatment with probiotics (*Lactobacillus helveticus* Rosell^®^-52 and *Bifidobacterium longum* Rosell^®^-175) or placebo consumed once daily [59]. Unblinding was permissible only if any serious adverse events occurred during the trial; however, it was not necessary. Randomization was performed using a computer-based random number generator (https://www.randomizer.org/, accessed on 10 December 2020).

The study population finally consisted of 60 patients recruited in psychiatric outpatient clinics in central Poland and through advertisements in social media. Regarding the sample size, the study was designed to be large enough to provide sufficient information on the chosen feasibility outcome measures [60]. As such, the recruitment goal for the internal pilot trial was 60 participants; however, we aimed to reach at least 9% of the sample size of the main planned trial for each study group [63].

A primary inclusion criterion was a diagnosis of depressive disorders, e.g., depressive episode, recurrent depression, mixed depressive and anxiety disorder [62], as this would provide a basis. The aim of this primary inclusion criterium was to provide the basis for performing a study on a real-life population with depression [64,65]. The remaining inclusion criteria comprised age above 18 years, the Montgomery-Asberg Depression Rating Scale (MADRS) score ≥ 13, and no change in antidepressant and antianxiety medications three weeks prior to the beginning of the study. The exclusion criteria comprised the following: pregnancy, an infection and/or vaccination and/or treatment with antibiotics in the previous four weeks, supplementation with probiotics or prebiotics in the previous four weeks, being diagnosed with or having new symptoms of autoimmune, serious immunocompromised, inflammatory bowel diseases, cancer, IgE-dependent allergy in the previous four weeks, a significant change in a dietary pattern in the previous four weeks, a significant change in dietary supplementation in the previous four weeks, a significant change in daily physical activity or an extreme sport activity in the previous four weeks, a significant change in a smoking pattern in the previous four weeks, a significant change in the treatment schema with proton-pump inhibitors, metformin, laxatives, systemic steroids, nonsteroidal anti-inflammatory drugs, antipsychotics, or any other medications influencing the microbiota according to present knowledge in the previous four weeks, current decompensated serious somatic disease, psychiatric comorbidities (except for a specific personality disorder, an additional specific anxiety disorder, and caffeine or nicotine addiction), a major neurological disorder or any medical disability that may interfere with a subject’s ability to complete the study procedures, high risk of suicide, and the current or recent participation in another research study involving an intervention that may alter outcomes that are relevant for this study. The criteria were constructed based on known factors influencing depressiveness, inflammation, metabolic or microbiota health states [66,67,68,69].

The study timeline has been previously described in detail [59].

### 2.2. Outcome Measures

The primary outcome measure was the rate of eligibility per month. The secondary outcome measures were rates of recruitment, enrolment per month, retention, and adherence, as well as capacity and resources to conduct all trial processes, or data completeness.

The rate of eligibility was assessed as the number of subjects eligible per month. Furthermore, an eligibility ratio was constructed to show the proportion of subjects who met the eligibility criteria to all those who were assessed for eligibility.

The recruitment rate was determined by the number of participants recruited for the first assessment meeting per month. The enrolment rate referred to the eligible participants who successfully enrolled in the study per month. The retention ratio was the proportion of subjects who completed the 60-day intervention period and were finally assessed to enrolled participants. Adherence was assessed as compliance with all study procedures: completing self-rated psychometric and dietary questionnaires on time, and a monitoring questionnaire every 15-days, the self-collection of stool samples, and the following of the inclusion and exclusion criteria, which was dependent on participants’ will. Later in the study, participants were also asked to confirm their regular intake of supplement capsules in the study’s daily medication chart.

Regarding feasibility, the capacity of clinicians to recruit and assess participants, the willingness of participants to be randomized, or the resources within the health care system and clinical setting to perform every trial procedure were considered [70].

The tertiary outcome measures included various demographic, diet- and health-related data, and selected clinical and laboratory parameters. Explanations for these outcome measures are provided in the protocol [59]. Seven of the outcome measures address clinical features, e.g., weight, waist circumference, blood pressure (BP), depressiveness, anxiety and stress symptoms, and quality of life (QoL). The laboratory outcome measures were C-reactive protein (CRP), complete blood count (CBC), specifically neutrophiles (NEU), lymphocytes (LYM) and platelets (PLT), alanine aminotransferase (ALT) and aspartate aminotransferase (AST), as well as the parameters included in International Diabetes Federation MetS criteria, i.e., fasting glucose (fGlc), high-density lipoprotein cholesterol (HDL-c) or triglicerides (TG) [71]. The indirect outcome measures calculated from the above included NEU/LYM, PLT/LYM, the systemic immune-inflammation index (SII; NEU*PLT/LYM), for the assessment of inflammation, and TG/HDL-c, AST/ALT, non-invasive markers of liver fibrosis: AST/PLT ratio index (APRI), Fibrosis-4 (FIB-4; (age*AST)/(PLT*√(ALT))) for the assessment of MetS complications risk.

### 2.3. Questionnaires and Scales

The characteristics of the questionnaires used may be found in the protocol [59].

Study-specific questionnaires were used: an initial study questionnaire assessing demographic, life-style and health-related data and the inclusion criteria for the study, and a monitoring questionnaire administered every 15 days to gain information on any adverse events or exclusion criteria emerging during the intervention period.

Validated scales were used to study the diet (the Food Frequency Questionnaire by Wądołowska [72]) and assess clinical outcome measures (the Montgomery-Asberg Depression Rating Scale (MADRS) [73], Depression, Anxiety and Stress Scale [74] and the WHO Quality of Life BREF Instrument [75]).

The questionnaires were provided as both editable electronic (sent by e-mail) and paper-and-pencil forms. This allowed the suitability of the methods for data collection to be compared.

### 2.4. Biological Material

Fasting venous blood was collected by qualified nurses (20 mL) in the morning, between 8:00 and 11:00 a.m., after overnight rest. One sample was collected at the beginning of the intervention period (V1) and another at the end (V2). The blood serum was frozen for future analyses. Stool samples were self-collected by study participants and were given to study investigators on the same or the next day after in-person meetings. Participants were given specific sterile containers for stool samples. If it was not possible to give the samples to study investigators within one hour of collection, the stool was to be kept frozen in any available freezer until the next day.

### 2.5. Intervention

At the beginning of the intervention period (V1), the participants were requested to follow their routine lifestyle activities over the following 60 days, and their observations were followed by a study-specific monitoring questionnaire. The probiotic group (PRO) received one capsule containing the probiotic mixture powder in the amount of 3 × 10^9^ CFU. The probiotic preparation contained *Lactobacillus helveticus* Rosell^®^-52, *Bifidobacterium longum* Rosell^®^-175 and excipients (Sanprobi Stress^®^, Sanprobi Sp. z o. o., Sp. k., Szczecin, Poland; probiotic powder manufacturer—Institute Rosell-Lallemand, Montreal, Canada). The placebo group (PLC) received the same capsule containing only the excipients (Sanprobi Sp. z o. o., Sp. k., Szczecin, Poland). The compliance with supplement intake was assessed with the monitoring questionnaire.

The optimal probiotics strains and dosages, intervention length and outcome measures were selected based on our previous investigation [47].

### 2.6. Data Management

The study used both self-administered and specialist-administered questionnaires completed as face-to-face interviews, paper-and-pencil tests, or, later in the study, on-line surveys. The samples included blood collected by qualified nurses and stool samples collected by the participants. Data entry was validated by the principal study investigator, and data review was performed by the co-investigators. The data was catalogued in a standardized way in compliance with the requirements of findability, accessibility, interoperability, and reusability (FAIR) standards and according to the General Data Protection Regulation (EU) 2016/679 (GDPR). All study participants provided their informed consent to data processing for one or more purposes. To ensure confidentiality, all patients were provided individual specific ID codes which were used to refer to data in the informed consent. The CC-BY-NC-ND 4.0 creative common license schema was applied.

### 2.7. Ethics

The study was conducted in accordance with the Declaration of Helsinki, and the principal study investigator gained approval from the Bioethics Committee of the Medical University of Lodz on 15 December 2020 (reference number RNN/228/20/KE).

### 2.8. Data Analysis

Statistical procedures were performed with STATISTICA 13.1 (TIBCO Software Inc., Tulsa, OK, USA). Descriptive statistics (means and standard deviations or means with 95% confidence interval) were generated for continuous variables. For discrete variables, the number of patients and percentages are given. Normality of distribution was tested with the Shapiro-Wilk test. The U-Mann Whitney test and the Kruskal-Wallis test were used to test inter-group differences. The repeated measures ANOVA was used to verify if any significant differences were found between variables over time. Associations were tested by Spearman’s correlation coefficients. The significance level was set at *p* < 0.05.

## 3. Results

### 3.1. Participant Flow

**Figure 1:**
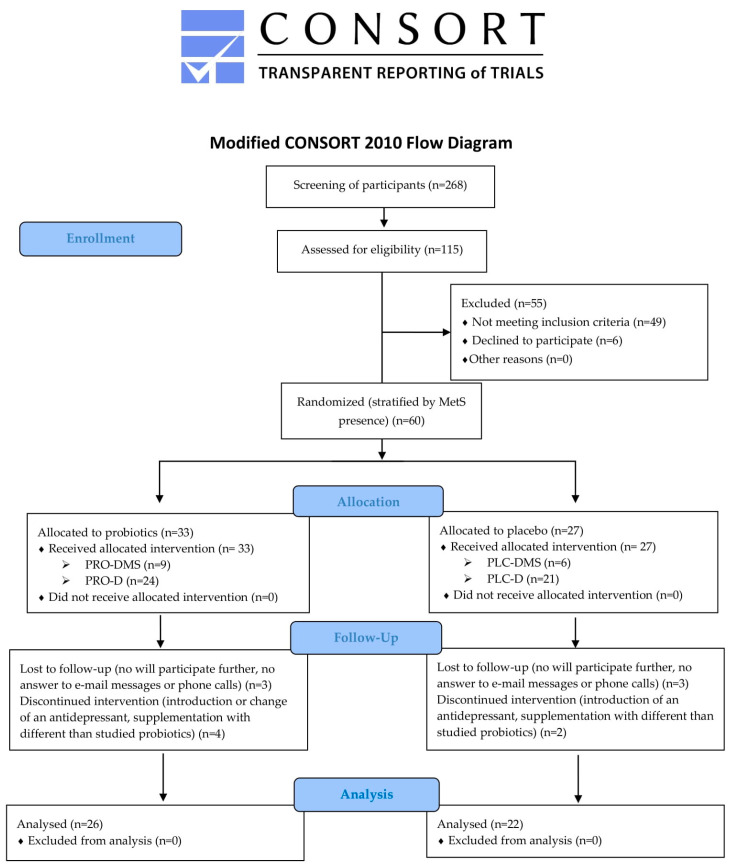
Participant flow diagram. MetS: metabolic syndrome; PRO-DMS: probiotic + depression + MetS group; PRO-D: probiotic + depression group; PLC-DMS: placebo + depression + MetS group; PLC-D: placebo + depression group.

Figure 1 shows the modified CONSORT flow diagram of study participants.

### 3.2. Feasibility

#### 3.2.1. Rate of Recruitment, Eligibility, Enrolment, and Retention

A rapid screening phase was first performed. The potential trial participants were familiarized with the list of eligibility criteria in electronic, paper or phone call form, together with information on the purpose and structure of the investigation. A total of 268 subjects were screened between December 2020 and December 2022, and 115 declared an interest in participating (43%). The mean recruitment rate was 4.8 participants per month, ranging from 0 to 27 participants. The main causes of not being recruited given by the subjects were not fulfilling the eligibility criteria (*n* = 68), living too far from the place of investigation (*n* = 12), not willing to follow the eligibility criteria thought the intervention period (*n* = 11), and not willing to be given the placebo intervention (*n* = 9). Fifty-three of the screened subjects did not provide any answer.

Following this, the inclusion and exclusion criteria were assessed by one of the study investigators during an on-line or in-person meeting. A total of 52% (60/115) of the assessed participants were eligible for enrolment. The enrollment ratio was 100%. The mean enrolment rate was 2.5 per month, ranging from 0 to 11 participants. Thirty-three subjects were allocated to be given the probiotic and 27 to be given the placebo intervention. As this pilot study is an internal part of the full Pro-demet RCT, randomization was performed for the full study, and the numbers of pilot trial participants were not necessarily equal. All participants received the allocated intervention.

#### 3.2.2. Retention Ratio, Intervention Adherence and Tolerability

The retention ratio was 80%, i.e., 78.8% in the probiotic group and 81.5% in the placebo group (*p* = 0.79). Three subjects in the PRO (two PRO-D and one PRO-DMS) as well as three subjects in PLC (all three PLC-D) group were lost to follow up. The reasons were the same in both groups: the participant was unwilling to continue the trial procedures and did not answer e-mail messages or phone calls. Four subjects in the PRO group discontinued intervention, two of them changed their antidepressant (both from PRO-D group), one from PRO-DMS group doubled the dose of an antidepressant, and one from the PRO-D group introduced supplementation with a different probiotic without a given reason. Two subjects from the PLC group (both PLC-D) discontinued intervention, one of them changed their antidepressant, and one suffered an acute infection and was treated with antibiotics and probiotics.

All participants reported full adherence and acceptability of the intervention over the 60 days, as shown in the monitoring questionnaire.

With regard to tolerability, no serious adverse events were observed. The participants in the PLC group reported acute upper airway infection (including COVID-19; *n* = 3) and urinary tract infection (*n* = 1), while those in the PRO group reported diarrhea (*n* = 2), upper airway infections (*n* = 1), and the exacerbation of allergic asthma (*n* = 1).

A statistical analysis was performed on data from 26 participants in the PRO group, and from 22 participants in the PLC group. However, due to the presence of confounders, some outcome measures were excluded from the analysis, e.g., inflammation parameters when acute infection symptoms were reported in the monitoring questionnaire administered at timepoint t3 or V2 (*n* = 6); depression, anxiety and stress score, or QoL measures when severe stressful life events or a fundamental change in life conditions were reported during V2 assessment (*n* = 4); and weight and waist circumference when supplementation of thyroid hormones was introduced (*n* = 1). One participant was unable to attend the in-person V2 meeting and only psychometric scores via the on-line visit were assessed. Finally, the psychometric scales score was analyzed from 44 participants, metabolic parameters from 46 participants, and inflammation parameters from 43 participants.

#### 3.2.3. Procedures

No problems were associated with randomization and blinding by an independent researcher. Nor were any problems found with regard to blood collection or anthropometric measures.

Regarding the self-assessment questionnaires, the content of the study-specific initial questionnaire and monitoring questionnaire appear reliable enough to gain all necessary information on potential confounders. However, additional questions were provided regarding any significant life events or changes that the participant believed influenced their mood or stress level. The self-assessment questionnaire collection had a strong influence on ensuring data completeness. The questionnaires were initially distributed in paper and electronic (via e-mail message) form. Unfortunately, several subjects (*n* = 14) did not return at least one of the questionnaires despite reminder messages and phone calls, resulting in some missing data. Therefore, the questionnaires were later issued as on-line tools, starting from subject number 50. Furthermore, to get additional data on the longitudinal assessment of self-rated symptoms, the DASS was subjected to additional timing with the monitoring questionnaire (starting from time point t3 of subject number 47).

Stool collection was generally complete, apart from three participants, one of whom lacked samples for both the V1 and V2 time points, and two of them were lost to follow up. In addition, several participants had to receive additional reminders for up to three consecutive days. It was subsequently decided to only give the investigated preparation to subjects that had completed both blood and feces samples collection.

Two study investigators were involved in the investigation period (recruitment, enrolment, data collection and entry). Finally, the principal study investigator was responsible for 34 study participants, and the auxiliary study investigator the other 14 subjects. It is planned to enlist further research staff to support the advertising and recruitment processes.

No problems were revealed for the analysis.

### 3.3. Population Characteristics

Approximately 27% of cases in the PRO and PLC groups were MetS subjects (see Table 1). Unfortunately, as this was too few for the study design, it was decided to compare the entire PRO and PLC groups regarding the sample characteristics.

The general characteristics of the study participants are shown in Table 1. Importantly, no differences in sociodemographic and general health-related parameters were found between the PLC and PRO groups at the beginning of the study. Specifically, the study participants did not differ significantly in terms of sex, age, main psychiatric diagnosis, psychotropic medications (e.g., antidepressants, antipsychotics), comorbidities, previously having COVID-19, different than psychotropic pharmacological treatment, frequency of cigarette or tobacco smoking, dietary supplement intake, being overweight or obese (BMI or waist circumference), or any other MetS components.

Additionally, dietary intake did not significantly differ between the two groups except for dairy and eggs (Table 2). However, this group has a lot of variation, including both natural and highly-processed foods, e.g., natural yoghurt or cottage cheese vs. sweet yoghurt or flavor-enhanced cottage cheese. Therefore, the intake of highly-processed foods was compared between the PRO and PLC groups. It was found that the groups did not differ with regard to their highly-processed dairy intake.

The PRO and PLC groups also demonstrated similar results for the psychometric questionnaire (Table 3), and for metabolic (Table 4) and inflammation (Table 5) markers at the start of the intervention period. This apparent lack of virtually any differences between the PLC and PRO groups represents an obvious strength of our study.

### 3.4. Clinical and Laboratory Outcome Measures

#### 3.4.1. Psychometric Measures

Psychometric results from 19 subjects in PLC (13 in PLC-D and 6 in PLC-DMS) and 25 subjects in PRO (19 in PRO-D and 6 in PRO-DMS) were analyzed. The mean changes between time-points V2 and V1 with 95% confidence intervals are shown in Table 6.

We performed an analysis of preliminary data gathered through this pilot study. While comparing groups regarding the MADRS score, the size effect was 0.51, and the power was 0.49. With this data, in order to achieve a power of 0.8 the study should involve approximately 240 subjects (60 in each subgroup).

#### 3.4.2. Metabolic Syndrome Components and Related Parameters

Anthropometric parameters, values of MetS criteria, and some additional laboratory metabolic health-related parameters results from 21 subjects in PLC (15 in PLC-D and 6 in PLC-DMS) and 25 subjects in the PRO group (19 in PRO-D and 6 in PRO-DMS) were analyzed. The mean changes between time-points V2 and V1 with 95% confidence intervals are shown in Table 7.

#### 3.4.3. Inflammation-Related Parameters

Laboratory inflammation-related parameters results from 19 subjects in PLC (14 in PLC-D and 5 in PLC-DMS) and 24 subjects in the PRO group (18 in PRO-D and 6 in PRO-DMS) were analyzed. The mean changes between time-points V2 and V1 with 95% confidence intervals are shown in Table 8.

## 4. Discussion

This study was aimed to assess the feasibility of a larger RCT. The rate of enrolment was shown to be 6.5 participants per month, which was lower than expected. Several obstacles in the process were identified; however, most of them were not modifiable. One problem was posed by the lockdown procedures associated with the COVID-19 pandemic [76], whose second wave occurred in Poland at the very beginning of the recruitment period (October 2020–January 2021). First of all, the safety procedures undertaken by medical services made it periodically impossible or very difficult to perform in-person meetings. Secondly, the fear of infection prevented potential participants from attending recruitment visits. Importantly, recruitment was also severely obstructed for four months in 2022 (July until September) because of procedural difficulties with laboratory availability for the trial. Thus, the most productive period of screening and recruitment was between October and December 2022, with a mean recruitment rate of 23 subjects per month. Overall, the findings demonstrate moderate recruitment potential, with the recruitment rate as expected or even higher when only considering working periods.

More than half (52%) of the recruited participants were found to be eligible and then enrolled. This was in accordance with our hypothesis. The main problem regarding the final study population nesting was the insufficient proportion of participants with MetS. This may be partly due to recruiting younger participants not yet fulfilling the MetS criteria, despite being overweight or suffering from abdominal obesity. In addition, part of the recruitment process took place through notices on social media, and, due to the pandemic, the major part of the assessment process was undertaken on-line; this may have discouraged middle-aged or older people. Additionally, older subjects might have been more afraid of COVID-19 infection.

It is not entirely surprising that a lower number of MetS patients was recruited than those without MetS, as only around one third of the population with depressive disorders suffer from comorbid MetS [77]. In a future full-scale study, we plan to advertise more via paper leaflets and to broaden the target locations from psychiatric clinics to include obesity, diabetes, cardiology or metabolic outpatient clinics; the internet advertising will also aim to include the social media groups of patients suffering from MetS and complications related to it.

The progression criteria, as previously outlined, was met, with the retention rate reaching the 80% threshold. This further confirms the feasibility of the full-scale RCT.

Missing data and dropouts are issues in most RCTs, and were shown to be crucial for our study’s feasibility and data completeness procedures. Therefore, some changes were introduced even during the pilot study, and these are thoroughly described in the Results section.

The pilot study hence raised several practical issues that can be addressed for the full RCT. In addition to those listed above, the study will incorporate a daily medication chart in paper form to improve the adherence to intervention. Moreover, various procedures, ranging from advertising the study to administering laboratory findings to study participants will be improved. For example, several ready-to-use text messages for different study stages have been designed in both paper and electronic form.

The baseline participant age, sex, comorbidities, smoking status or anthropometric measures are similar to those reported in other studies investigating probiotics in populations with depressive disorders [49,57,58]. Importantly, there was only one difference between the probiotic and placebo group at the beginning of the intervention period, namely dairy and eggs intake. However, when the intake of highly-processed dairy products was considered, the difference was no longer significant.

A change in tertiary outcome measures were observed between time-points V1 and V2. All changes were given 95% confidence intervals. Interestingly, the mean MADRS score change was −5.47 points in PRO-D but only −2.54 in PLC-D; it has been suggested that an improvement of 2 points or more on the MADRS may be clinically relevant [78,79]. Furthermore, the total QoL score was found to increase by 11.73 points in PRO-D and 6.00 in PLC-D; similar changes were found in the psychological subscale score. In addition, the CRP level decreased by 0.64 mg/L in PRO-D and increased by 0.27 mg/L in PLC-D. Nonetheless, 95% CI and insufficient power need to be considered when interpreting the results.

Pilot trials are not primarily interested in treatment effect or efficacy. Indeed, according to the CONSORT statement (extension to randomized pilot and feasibility trials), formal hypothesis testing for effectiveness is not recommended in a pilot trial [61]. As the aim of a pilot trial is not to assess efficacy, and our pilot study is underpowered in this regard anyway, it does not discuss the changes in parameters between V1 and V2 time-points in detail (Table 6, Table 7 and Table 8).

The future full-scale RCT should avoid sources of bias that might influence the estimation of treatment effect, including selection or attrition bias. Fortunately, our pilot findings indicate that this is highly likely.

The limitations of the study include the low proportion of MetS participants and lack of full data completeness regarding self-assessment questionnaires; however, these will be addressed in the future definitive RCT. Additionally, it was not possible to fully evaluate the tertiary outcome measures due to the small number of participants meeting the criteria for MetS. Nonetheless, it was not a primary or even secondary aim of this pilot study. The study also has some important strengths, mainly the high levels of acceptability, good retention ratio, and the detailed description of enrolled participants. Another strength is its selection of outcome measures, which covers several potential areas of probiotic action.

## 5. Conclusions

In conclusion, this pilot study confirms the feasibility of its subsequent full-scale RCT investigating the effect of probiotics in the treatment of depressive disorders with possible comorbid MetS and its components. Hence, the study would be suitable for determining the potential clinical use of probiotics and assessing certain key factors such as potential biomarkers of response. Although our manuscript only addresses the procedures performed in a single pilot trial, we hope that the methods may be useful for planning and analyzing other pilot studies.

No previous study has examined the effects of probiotic supplementation on psychometric parameters together with the metabolic profile, serum inflammation markers, and biomarkers of MAFLD in patients with depressive disorders in general. The full-scale Pro-demet RCT is intended to evaluate the effects of two-strain probiotic intake on the above parameters in a real-life population with depressive disorders.

## Figures and Tables

**Table 1:** General characteristics of the study participants at the start of the trial.

Characteristics	PRO Group (*n* = 26)	PLC Group (*n* = 22)	*p*
Sex (F:M)	21:5	20:2	0.56
Age (years)	34.30	35.70	0.37
Diagnosis according to ICD-11 (6A70:6A71:6A72:6A73)	5:9:0:12	4:2:0:16	0.13
Psychotropic medications (%)	61.5	81.8	0.13
Antidepressants (%)	61.5	81.8	0.13
Antipsychotics (%)	11.5	4.5	0.73
Comorbidities (%)	38.5	54.5	0.27
COVID-19 in the past (%)	15.4	18.2	0.95
Other than psychotropics pharmacological treatment (%)	34.6	45.5	0.45
Smoking cigarettes (%)	19.2	22.7	0.77
Dietary supplements (%)	57.7	36.4	0.14
Overweight (according to BMI) (%)	38.5	31.8	0.58
Obesity (according to BMI) (%)	11.5	22.7	
MetS (%)	26.9	27.3	0.97
Abdominal obesity (%)	53.8	63.6	0.49
Raised TG (%)	11.5	18.2	0.81
Reduced HDL-c (%)	19.2	13.6	0.89
Raised BP (%)	46.2	40.9	0.72
Raised fGlc (%)	15.4	13.6	0.81

F: females; M: males; 6A70: single episode depressive disorder; 6A71: recurrent depressive disorder; 6A72: dysthymic disorder; 6A73: mixed depressive and anxiety disorder; BMI: body mass index; MetS: metabolic syndrome; TG: triglycerides; HDL-c: high-density lipoproteins cholesterol; BP: blood pressure; fGlc: fasting glucose.

**Table 2:** Dietary intakes of study participants at the beginning of the study according to Food Frequency Questionnaire-6.

Characteristics	PRO Group	PLC Group	*p*
Sweets and snacks	2.42 ± 0.52	2.85 ± 0.85	0.09
Dairy and eggs	2.81 ± 0.61	3.21 ± 0.86	0.01
Highly-processed	1.58 ± 0.57	2.12 ± 1.09	0.16
Cereal products	3.04 ± 0.54	3.20 ± 0.52	0.20
Oils	2.48 ± 0.71	2.55 ± 0.67	0.48
Fruits	2.65 ± 0.48	2.63 ± 0.51	0.66
Vegetables and seeds	3.27 ± 0.45	3.42 ± 0.70	0.41
Meat (including fish)	2.40 ± 0.64	2.29 ± 0.52	0.44
Drinks (excluding water)	2.14 ± 0.55	1.95 ± 0.52	0.22
Highly-processed food products	2.29 ± 0.42	2.44 ± 0.34	0.17

Food frequency intake assessed in a scale 1–6: 1—never or almost never; 2—once a month; 3—several times a month; 4—several times a week; 5—every day; 6—several times a day.

**Table 3:** Psychometric scores of the study participants at the beginning of the study.

Characteristics	PRO Group	PLC Group	*p*
MADRS score	20.12 ± 5.38	17.7 ± 4.08	0.17
DASS score	61.24 ± 21.66	57.93 ± 19.71	0.58
Depression	20.41 ± 11.43	20.53 ± 8.84	0.88
Anxiety	16.59 ± 7.98	14.83 ± 6.85	0.55
Stress	24.24 ± 8.82	22.67 ± 10.36	0.60
QoL score	71.69 ± 12.88	73.07 ± 12.97	0.76
Physical	17.88 ± 4.69	19.23 ± 4.12	0.34
Psychological	13.75 ± 3.02	15.29 ± 4.14	0.29
Social	9.35 ± 2.26	8.00 ± 1.97	0.12
Environment	25.71 ± 4.96	25.71 ± 4.96	0.25

MADRS: Montgomery–Åsberg Depression Rating Scale; DASS: Depression, Anxiety and Stress Scale; QoL: quality of life.

**Table 4:** Metabolic parameters of the study participants at the beginning of the study.

Characteristics	PRO Group	PLC Group	*p*
Weight (kg)	71.95 ± 17.26	70.92 ± 17.26	0.99
BMI (kg/m^2^)	24.29 ± 3.41	25.77 ± 5.98	0.49
WC * (cm)	86.46 ± 11.76	86.60 ± 15.85	0.90
sBP * (mmHg)	122.48 ± 16.77	120.19 ± 18.22	0.53
dBP * (mmHg)	83.28 ± 10.22	81.52 ± 9.38	0.66
fGlc * (mmol/l)	5.22 ± 0.54	5.09 ± 0.44	0.53
HDL-c * (mmol/l)	1.67 ± 0.46	1.56 ± 0.30	0.43
TG * (mmol/l)	1.19 ± 0.68	1.35 ± 0.52	0.10
TG/HDL-c	0.80 ± 0.56	0.95 ± 0.48	0.11
AST (U/l)	24.69 ± 6.17	24.27 ± 10.01	0.44
ALT (U/l)	22.52 ± 15.79	21.28 ± 16.28	0.83
AST/ALT	1.36 ± 0.49	1.32 ± 0.41	0.95
APRI	0.27 ± 0.10	0.25 ± 0.10	0.29
FIB-4	0.73 ± 0.38	0.72 ± 0.36	0.93

BMI: body mass index; WC: waist circumference; sBP: systolic blood pressure; dBP: diastolic blood pressure; fGlc: fasting glucose; HDL-c: high-density lipoprotein cholesterol; TG: triglicerides; AST: aspartate aminotransferase; ALT: alanine aminotransferase; APRI: AST/PLT ratio index; FIB-4: Fibrosis-4; *: metabolic syndrome criteria components.

**Table 5:** Inflammation parameters of the study participants at the beginning of the study.

Characteristics	PRO Group	PLC Group	*p*
CRP (mg/L)	2.37 ± 1.84	2.54 ± 2.55	0.65
WBC (×10^3^/µL)	5.79 ± 1.31	6.25 ± 1.45	0.30
NEU (×10^3^/µL)	3.13 ± 1.05	3.37 ± 1.03	0.39
LYM (×10^3^/µL)	1.88 ± 0.55	2.12 ± 0.49	0.09
PLT (×10^3^/µL)	278.12 ± 64.99	282.00 ± 48.51	0.34
NEU/LYM	1.87 ± 1.26	1.64 ± 0.51	0.89
PLT/LYM	158.10 ± 47.19	137.42 ± 34.34	0.06
SII	507.23 ± 305.66	455.90 ± 148.14	0.89

CRP: C-reactive protein; WBC: white blood cells; NEU: neutrophils; LYM: lymphocytes; PLT: platelets; SII: systemic inflammatory index.

**Table 6:** Change in psychometric scores of the study participants.

Characteristics [mean ± SD]	V1 PRO-D	V2 PRO-D	Δ [95% CI]	V1 PLC-D	V2 PLC-D	Δ [95% CI]	V1 PRO-DMS	V2 PRO-DMS	Δ [95% CI]	V1 PLC-DMS	V2 PLC-DMS	Δ [95% CI]
MADRS score	20.11 ± 5.21	14.63 ± 5.19	−5.47 [−8.12, –2.81]	17.15 ± 4.06	14.62 ± 6.19	−2.54 [−5.57, 0.49]	20.17 ± 6.40	19.67 ± 5.61	−0.50 [−5.50, 4.50]	19.00 ± 4.20	14.83 ± 6.55	−4.17 [−8.83, 0.50]
DASS score	62.25 ± 17.43	45.75 ± 20.12	−16.50 [−31.10, −1.90]	55.50 ± 17.14	41.00 ± 23.23	−14.50 [−25.67′ −3.33]	58.80 ± 32.10	54.00 ± 17.68	−4.80 [−32.07, 22.47]	62.80 ± 25.58	50.20 ± 24.63	−12.6 [−32.52, 7.32]
Depression	19.25 ± 10.12	15.00 ± 9.76	−4.25 [−9.84, 1.34]	19.60 ± 8.95	15.10 ± 8.74	−4.50 [−9.52, 0.52]	23.20 ± 15.07	21.60 ± 11.99	−1.60 [−10.62, 7.42]	22.40 ± 9.32	20.40 ± 10.67	−2.00 [−9.24, 5.24]
Anxiety	16.33 ± 7.24	10.08 ± 6.58	−4.25 [−9.84, 1.34]	14.30 ± 7.07	9.50 ± 7.79	−4.50 [−9.52, 0.52]	17.20 ± 10.47	13.20 ± 3.77	−1.60 [−10.62, 7.42]	15.60 ± 7.09	11.60 ± 5.55	−2.00 [−9.24, 5.24]
Stress	26.67 ± 6.91	20.67 ± 10.29	−6.00 [−13.58, 1.58]	21.60 ± 10.29	16.40 ± 10.75	−5.20 [−10.18, −0.22]	18.40 ± 10.95	19.20 ± 9.04	0.80 [−8.95, 10.55]	24.80 ± 11.34	18.20 ± 9.28	−6.60 [−16.86, 3.66]
QoL score	71.73 ± 13.44	83.45 ± 9.41	11.73 [0.35, 23.11]	76.70 ± 13.05	82.70 ± 15.23	6.00 [−0.23, 12.23]	71.60 ± 13.05	69.80 ± 12.54	−1.80 [−13.69, 10.09]	64.00 ± 8.04	66.25 ± 9.43	2.25 [−1.93, 6.43]
Physical	17.18 ± 5.17	20.73 ± 2.53	3.55 −0.22, 7.31]	20.00 ± 4.28	23.00 ± 4.08	3.00 [0.57, 5.43]	19.17 ± 3.71	20.00 ± 6.78	0.83 [−4.20, 5.86]	16.75 ± 2.50	16.25 ± 2.36	−0.50 [−4.71, 3.71]
Psychological	13.73 ± 3.26	16.82 ± 3.25	3.09 [−0.01, 6.19]	16.50 ± 3.92	17.40 ± 4.17	0.90 [−0.87, 2.67]	13.80 ± 2.77	13.80 ± 2.86	0.00 [−1.76, 1.76]	12.25 ± 3.30	13.00 ± 3.46	0.75 [−0.77, 2.27]
Social	9.55 ± 2.25	10.18 ± 1.83	0.64 [−0.65, 1.92]	8.38 ± 2.02	9.69 ± 1.97	1.31 [0.44, 2.18]	9.00 ± 2.45	9.83 ± 2.48	0.83 [−0.97, 2.64]	6.75 ± 1.26	7.00 ± 1.41	0.25 [0.55, 1.05]
Environment	26.00 ± 4.43	29.27 ± 3.50	3.27 [−0.46, 7.00]	23.62 ± 5.84	26.85 ± 6.38	3.23 0.48, 5.98]	25.27 ± 6.24	25.67 ± 6.56	0.50 [−4.88, 5.88]	24.35 ± 2.63	25.50 ± 4.36	1.25 [−2.03, 4,53]

PRO-DMS: probiotic + depression + MetS group; PRO-D: probiotic + depression group; PLC-DMS: placebo + depression + MetS group; PLC-D: placebo + depression group; MADRS: Montgomery–Åsberg Depression Rating Scale; DASS: Depression, Anxiety and Stress Scale; QoL: quality of life.

**Table 7:** The values of metabolic health-related parameters at the beginning (V1) and the end (V2) of the pilot study. PRO-DMS: probiotic + depression + MetS group; PRO-D: probiotic + depression group; PLC-DMS: placebo + depression + MetS group; PLC-D: placebo + depression group.

Parameter [mean ±SD]	V1 PRO-D	V2 PRO-D	Δ [95% CI]	V1 PLC-D	V2 PLC-D	Δ [95% CI]	V1 PRO-DMS	V2 PRO-DMS	Δ [95% CI]	V1 PLC-DMS	V2 PLC-DMS	Δ [95% CI]
Weight (kg)	67.24 ± 10.55	67.71 ± 11.25	0.46 [−0.14, 1.06]	63.04 ± 12.95	63.28 ± 12.82	0.24 [−0.53, 1.01]	86.85 ± 21.66	85.83 ± 19.56	−1.02 [−4.08, 2.05]	89.32 ± 10.79	89.08 ± 11.23	−0.23 [−2.57, 2.11]
BMI (kg/m^2^)	23.29 ± 2.65	23.54 ± 2.86	0.13 [−0.08, 0.34]	23.05 ± 4.46	23.13 ± 4.35	0.08 [−0.20, 0.36]	28.07 ± 3.54	27.73 ± 2.95	−0.34 [−1.53, 0.85]	32.12 ± 3.86	31.37 ± 3.96	−0.03 [−1.18, 1.11]
WC (cm)	82.63 ± 9.90	82.03 ± 10.31	−0.60 [−2.32, 1.11]	79.79 ± 13.04	79.93 ± 12.85	0.14 [−1.59, 1.88]	98.58 ± 8.90	99.00 ± 9.84	0.42 [−2.20, 3.04]	102.50 ± 8.87	101..92 ± 7.53	−0.58 [−7.29, 6.12]
sBP (mmHg)	121.00 ± 16.69	121.37 ± 17.22	0.37 [−2.62, 3.36]	116.07 ± 14.41	117.87 ± 11.58	1.80 [−3.59, 7.19]	127.17 ± 17.66	124.50 ± 20.86	−2.67 [−7.44, 2.10]	130.50 ± 23.82	133.67 ± 14.00	3.17 [−10.11, 16.45]
dBP (mmHg)	82.47 ± 9.91	81.00 ± 7.98	−1.47 [−4.91, 1.97]	79.60 ± 8.36	79.60 ± 7.59	0.00 [−3.50, 3.50]	85.83 ± 11.72	85.17 ± 13.00	−0.67 [−4.85, 3.51]	86.33 ± 10.86	86.67 ± 10.93	0.33 [−5.36, 6.03]
fGlc (mmol/L)	5.18 ± 0.59	5.10 ± 0.48	−0.10 [−0.36, 0.15]	4.92 ± 0.31	4.91 ± 0.46	−0.02 [−0.21, 0.17]	5.36 ± 0.32	5.27 ± 0.39	−0.09 [−0.25, 0.07]	5.51 ± 0.46	5.55 ± 0.59	0.04 [−0.63, 0.71]
HDL-c (mmol/L)	1.75 ± 0.45	1.71 ± 0.37	0.07 [0.08, 0.22]	1.61 ± 0.28	1.68 ± 0.28	−0.14 [−0.32, 0.04]	1.42 ± 0.44	1.47 ± 0.45	−0.45 [−1.09, 0.19]	1.44 ± 0.34	1.38 ± 0.29	0.53 [−0.50, 1.56]
TG (mmol/L)	0.96 ± 0.29	1.03 ± 0.38	−0.04 [−0.20, 0.11]	1.24 ± 0.44	1.09 ± 0.42	0.06 [−0.03, 0.16]	1.92 ± 1.05	1.47 ± 0.56	0.05 [−0.07, 0.16]	1.65 ± 0.62	2.18 ± 1.15	−0.06 [−0.21, 0.09]
TG/ HDL-c	0.60 ± 0.29	0.63 ± 0.27	0.04 [−0.09, 0.17]	0.81 ± 0.35	0.69 ± 0.33	−0.12 [−0.25, 0.01]	1.41 ± 0.76	1.05 ± 0.45	−0.36 [−0.81, 0.09]	1.40 ± 0.60	1.65 ± 0.84	0.46 [−0.40, 1.33]
AST (U/L)	24.76 ± 5.69	23.51 ± 4.96	−1.25 [−3.37, 0.86]	21.98 ± 8.26	21.31 ± 6.31	−0.67 [−2.54, 1.19]	24.47 ± 8.14	24.58 ± 8.16	0.12 [−10.33, 10.56]	29.98 ± 12.39	26.80 ± 12.22	−3.18 [−6.65, 0.28]
ALT (U/L)	20.71 ± 10.71	19.33 ± 8.52	−1.38 [−4.24, 1.47]	15.71 ± 6.38	17.12 ± 7.16	1.41 [−1.15, 3.98]	28.25 ± 27.06	26.93 ± 26.37	−1.32 [−12.77, 10.14]	35.20 ± 24.91	34.02 ± 27.66	−1.18 [−9.64, 7.27]
AST/ ALT	1.38 ± 0.47	1.40 ± 0.60	0.03 [−0.13, 0.19]	1.47 ± 0.38	1.31 ± 0.30	−0.16 [−0.30, −0.02]	1.31 ± 0.61	1.35 ± 0.70	0.04 [−0.54, 0.61]	0.97 ± 0.28	0.98 ± 0.39	0.01 [−0.14, 0.16]
APRI	0.27 ± 0.08	0.26 ± 0.08	−0.01 [−0.04, 0.02]	0.23 ± 0.08	0.23 ±0.07	0.005 [−0.02, 0.03]	0.28 ± 0.16	0.27 ± 0.14	−0.01 [−0.15, 0.13]	0.31 ± 0.14	0.26 ± 0.12	−0.05 [−0.14, 0.03]
FIB-4	0.72 ± 0.39	0.75 ± 0.41	0.02 [−0.03, 0.08]	0.69 ± 0.34	0.66 ± 0.32	−0.03 [−0.09, 0.03]	0.74 ± 0.39	0.82 ± 0.65	0.075 [−0.23, 0.38]	0.78 ± 0.44	0.65 ± 0.25	−0.13 [−0.38, 0.11]

PRO-DMS: probiotic + depression + MetS group; PRO-D: probiotic + depression group; PLC-DMS: placebo + depression + MetS group; PLC-D: placebo + depression group; BMI: body mass index; WC: waist circumference; sBP: systolic blood pressure; dBP: diastolic blood pressure; fGlc: fasting glucose; HDL-c: high-density lipoprotein cholesterol; TG: triglycerides; AST: aspartate aminotransferase; ALT: alanine aminotransferase; APRI: AST/PLT ratio index; FIB-4: Fibrosis-4.

**Table 8:** The values of inflammation-related parameters at the beginning (V1) and the end (V2) of the pilot study.

Parameter [mean ±SD]	V1 PRO-D	V2 PRO-D	Δ [95% CI]	V1 PLC-D	V2 PLC-D	Δ [95% CI]	V1 PRO-DMS	V2 PRO-DMS	Δ [95% CI]	V1 PLC-DMS	V2 PLC-DMS	Δ [95% CI]
CRP (mg/L)	2.17 ± 1.88	1.53 ± 1.24	−0.64 [−1.66, 0.38]	1.91 ± 2.34	2.18 ± 2.03	0.27 [−0.59, 1.13]	2.97 ± 1.72	2.53 ± 1.98	−0.43 [−1.51, 0.65]	4.32 ± 2.48	3.88 ± 2.04	−0.44 [−5.18, 4.30]
WBC (×10^3^/µL)	5.88 ± 1.41	5.99 ± 1.51	0.11 [−0.44, 0.66]	5.87 ± 1.07	6.13 ± 1.19	0.26 [−0.25, 0.77]	5.52 ± 1.02	5.63 ± 1.53	0.11 [−0.57, 0.79]	7.29 ± 1.97	7.41 ± 1.72	0.12 [−1.49, 1.72]
NEU (×10^3^/µL)	3.23 ± 1.16	3.29 ± 1.15	0.05 [−0.42, 0.52]	3.06 ± 0.65	3.29 ± 0.73	0.23 [−0.19, 0.66]	2.80 ± 0.58	2.94 ± 0.85	0.14 [−0.19, 0.465]	4.22 ± 1.48	4.13 ± 0.99	−0.09 [−1.19, 1.01]
LYM (×10^3^/µL)	1.83 ± 0.57	1.93 ± 0.55	0.09 [−0.07, 0.26]	2.09 ± 0.51	2.13 ± 0.54	0.04 [−0.20, 0.28]	2.04 ± 0.48	1.97 ± 0.54	−0.06 [−0.35, 0.23]	2.23 ± 0.47	2.37 ± 0.64	0.15 [−0.45, 0.75]
NEU /LYM	2.02 ± 1.42	1.81 ± 0.74	−0.20 [−0.78, 0.38]	1.55 ± 0.49	1.63 ± 0.43	0.08 [−0.20, 0.36]	1.43 ± 0.34	1.51 ± 0.26	0.09 [−0.06, 0.23]	1.91 ± 0.50	1.80 ± 0.38	−0.11 [−0.68, 0.46]
PLT	271.47 ± 43.75	259.11 ± 47.68	−12.37 [−22.42, −2.32]	279.27 ± 45.05	275.53 ± 56.05	−3.73 [−18.42, 10.95]	299.17 ± 112.62	303.33 ± 105.85	4.17 [−15.34, 23.67]	288.83 ± 60.42	305.33 ± 39.62	16.50 [−23.35, 56.35]
PLT /LYM	161.27 ± 48.84	145.99 ± 48.43	−15.29 [−36.77, 6.20]	142.14 ± 38.89	136.14 ± 30.37	−6.00 [−23.41, 11.41]	148.57 ± 44.56	158.29 ± 43.53	9.72 [−18.29, 37.72]	124.22 ± 9.79	130.14 ± 34.21	5.92 [−38.95, 50.79]
SII	541.55 ± 344.05	482.12 ± 235.55	−59.42 [−199.08, 80.23]	432.21 ± 136.94	448.70 ± 133.64	16.49 [−70.82, 103.79]	404.27 ± 101.82	439.22 ± 82.38	34.95 [−0.55, 70.45]	522.24 ± 174.18	521.77 ± 104.44	−0.47 [−227.57, 226.62]

PRO-DMS: probiotic + depression + MetS group; PRO-D: probiotic + depression group; PLC-DMS: placebo + depression + MetS group; PLC-D: placebo + depression group; CRP: C-reactive protein; WBC: white blood cells; NEU: neutrophils; LYM: lymphocytes; PLT: platelets; SII: systemic inflammatory index.

## Data Availability

The data that support the findings of this study are available from the corresponding author, O.G.-K., upon reasonable request.
